# Association between severe lumbar disc degeneration and end-stage hip or knee osteoarthritis requiring joint replacement surgery: a population-based cohort study with a 26-year follow-up

**DOI:** 10.1007/s00402-025-05908-7

**Published:** 2025-05-12

**Authors:** Sami Salo, Reijo Sund, Toni Rikkonen, Jukka Huopio, Heidi Hurri, Heikki Kröger, Joonas Sirola

**Affiliations:** 1 Kuopio Musculoskeletal Research Unit (KMRU), Surgery, Institute of Clinical Medicine, University of Eastern Finland (UEF), P.O. Box 1627, FI-70211, Kuopio, Finland; 2 Department of Orthopaedics, Traumatology, and Hand Surgery, North Savo Wellbeing Services County, Kuopio University Hospital (KUH), Puijonlaaksontie 2, 70210, Kuopio, Finland

**Keywords:** Intervertebral disc degeneration, Osteoarthritis, Arthroplasty, Magnetic resonance imaging, Lumbar spine

## Abstract

**Introduction:**

An association between intervertebral disc degeneration (IDD) and osteoarthritis (OA) of the hip and knee has been found previously. However, studies using MRI-evaluated IDD grades with large sample sizes are lacking. Total hip and knee arthroplasty (THA and TKA) can be considered an indicator of clinically evident end-stage OA.

**Materials and methods:**

The study population consisted of 1,153 postmenopausal Finnish women with clinical back problems, of whom 166 had THA and 295 had TKA during the 26-year follow-up. The study was based on the prospective OSTPRE cohort established in 1989 and Finnish Arthroplasty Register data. The IDD severity was graded from T2-weighted lumbar MRI images using the five-grade Pfirrmann classification. Five intervertebral levels (L1-L2 to L5-S1) were studied (5,765 discs). Cox regression with a time-dependent covariate was used to calculate hazard ratios (HRs) for THA and TKA to account for exposure time for severe degeneration.

**Results:**

A higher hazard for THA was observed in women with severe IDD at the L1-L2 (HR 2.66, 95% CI 1.48–4.80), L2-L3 (HR 1.97, CI 1.15–3.38), L5-S1 (HR 1.69, 95% CI 1.16–2.46) and the L1-S1 (HR 2.19, 95% CI 1.53–3.15) intervertebral levels. Adjustment with potential confounders did not alter the results. Women with severe IDD had an elevated hazard for TKA at the L1-S1 mean degeneration (HR 1.50, CI 1.11–2.02) analysis. However, in the adjusted model, the statistical significance of this association was lost (HR 1.34, 95% CI 0.98–1.84). Higher BMI increased the hazard for both THA and TKA; however, the effect was more substantial for TKA.

**Conclusions:**

The present study supports the association between lumbar IDD and hip OA. A weak association between lumbar IDD and knee OA was observed. Further research is needed to investigate the causality of the relationship between IDD and OA.

**Supplementary Information:**

The online version contains supplementary material available at 10.1007/s00402-025-05908-7.

## Introduction

Osteoarthritis (OA) and low back pain (LBP) are the leading musculoskeletal causes of disability worldwide [1–3]. Intervertebral disc degeneration (IDD) has been linked to both LBP [4–6] and recurrent episodes of LBP [7]. However, it is essential to note that IDD does not always lead to LBP, and degenerative changes in the spine are also prevalent among asymptomatic individuals [8–10]. OA frequently occurs in the hip, knee, and spine but less often in the ankle, wrist, elbow, and shoulder [11]. Conservative treatment of OA includes, for example, physiotherapy, weight loss, and pharmacological therapy [12]. Despite conservative treatment, many patients with severe OA ultimately require total joint replacement, which can be considered an indicator of symptomatic end-stage OA [13]. Therefore, patients who have undergone total hip arthroplasty (THA) or total knee arthroplasty (TKA) can be considered to have suffered from clinically evident and symptomatic OA.

Previous studies have found an association between IDD and OA of the hip and knee [11, 14, 15]. However, inconsistent results have been reported, as knee OA has been found to be associated with osteophytes and facet joint OA but not with disc space narrowing, but no associations were found between hip OA and components of spine degeneration [16]. It has been suggested that lumbar disc degeneration precedes hip degeneration and may play a causal role in hip OA [11]. Radiographic lumbar spine degeneration and lumbar spine symptoms are common among patients with severe knee OA undergoing TKA [15]. A recent Mendelian randomization study demonstrated a bidirectional causal relationship between hip OA and IDD; however, a direct causal link between hip OA and IDD was considered to remain uncertain [17]. Furthermore, no significant association was found between knee OA and IDD [17]. OA burden, characterized by either previous THA or TKA, was recently shown to be independently associated with the severity of the IDD in a cohort of patients with degenerative lumbar spondylolisthesis [18].

The intervertebral disc and articular joint composition and process of degeneration are remarkably similar, and parallel cell-level mechanisms have been found in the development of both OA and IDD [19]. Several genetic factors attributed to both IDD and OA have also been described [20]. Mechanical overloading is a key factor in the development of both IDD and OA [19]. Sagittal pelvic morphology and spinopelvic-femoral dynamics may play an essential role in the development of IDD and OA. High pelvic incidence (PI) has been found to be a risk factor for the development of spondylolisthesis and knee OA [21]. Furthermore, low PI has been linked with degenerative disc disease (DDD) and may be associated with hip OA [21].

However, studies with large sample sizes investigating the connection between OA and IDD, evaluated using Magnetic Resonance Imaging (MRI), are lacking. Therefore, the present study aimed to investigate the association between MRI-evaluated lumbar IDD and clinically evident hip and knee OA, indicated with THA/TKA, in Finnish postmenopausal women. We hypothesized that severe IDD is associated with increased hip and knee arthroplasty hazard.

## Materials and methods

### Study design and setting

The present study was based on the prospective Kuopio Osteoporosis Risk Factor and Prevention (OSTPRE) study cohort, which was established in February 1989 by selecting all women born between 1932 and 1941 living then in the Province of Kuopio in Eastern Finland (*N* = 14,220). A baseline questionnaire was mailed to 14,220 participants in 1989. A total of 13,100 women responded to the questionnaire, which included questions about medical conditions, anthropometric measures, and other health-related factors. The study protocol has been described previously [22, 23]. Data on all TKAs and THAs in the OSTPRE study population were obtained from the Care Register for Health Care (CRHC) and the Finnish Arthroplasty Register (FAR), which together have been found to be more accurate than one register alone [24]. The CRHC contains all special healthcare hospital admissions in Finland. Arthroplasty operation records have been included in CRHC since 1986. The FAR has recorded data from arthroplasties in Finland since 1 January 1980 [25]. The data for this study were available and collected until 31 December 2016. The indications related to performed arthroplasties were obtained from the register data.

### Lumbar MRI scans

MRI data was obtained from the Kuopio University Hospital (KUH) image database, PACS (Picture Archiving and Communication System, Sectra, Sweden). PACS has been available at KUH since 2002. The lumbar spine MRI scans were performed between January 2003 and December 2015. All scans were performed with a 1.5 T MRI scan unit due to clinical indications, such as LBP, spinal stenosis, and neurological symptoms of the lower legs, among others. Indication or diagnosis related to lumbar MRI scan was also obtained from CRHC by observing the closest recorded diagnosis pertaining to the MRI imaging. Some of the women had several MRI scans during the follow-up period. In this case, the MRI scan of each woman was used to evaluate the severity of IDD.

### Study population

**Fig. 1:**
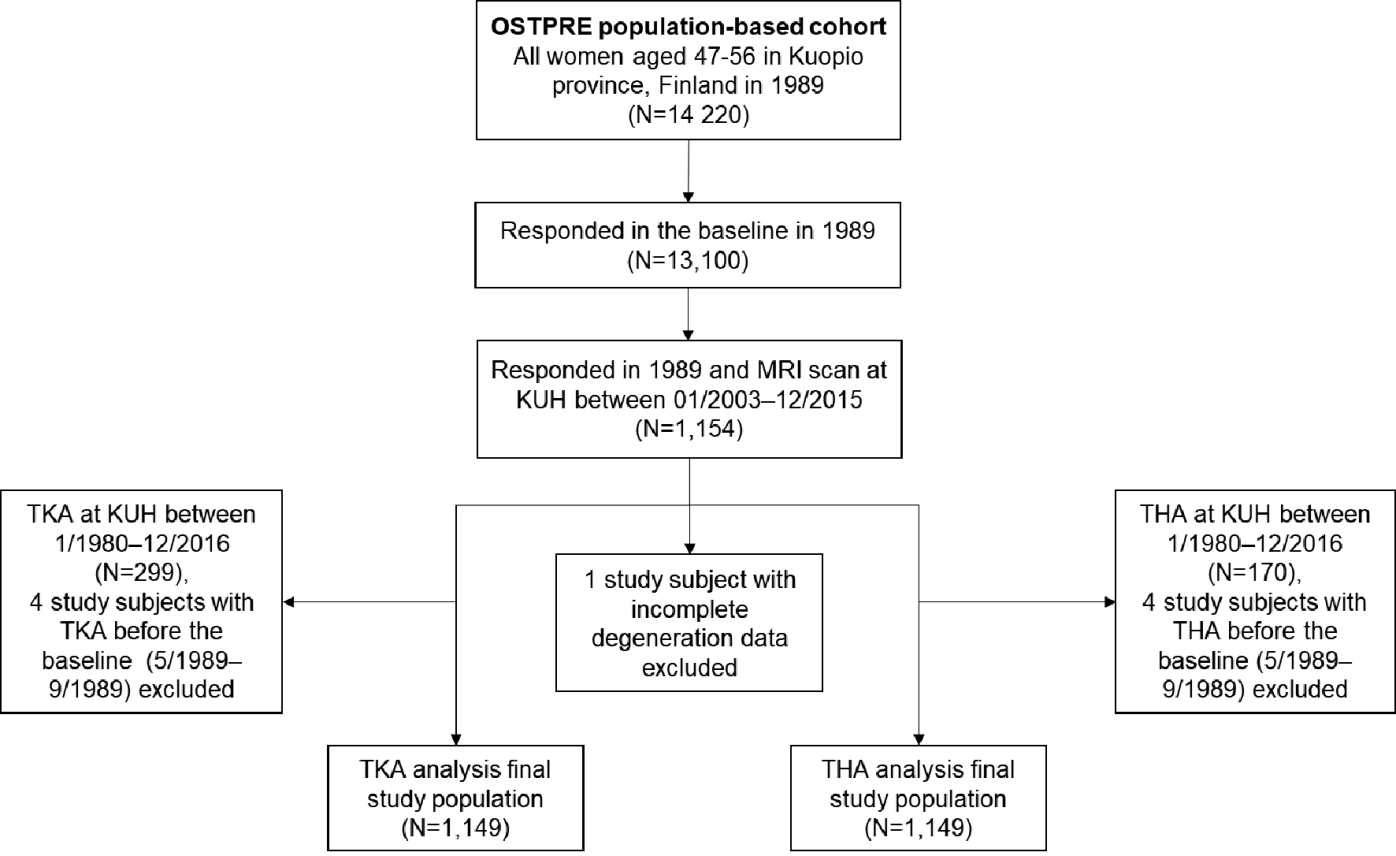
Selection of the study population

Altogether, 1,154 out of 13,100 OSTPRE baseline respondents had a lumbar MRI scan at KUH between January 2003 and December 2015. One study subject had an invalid degeneration classification and was excluded. Valid data on height, weight, medical conditions, and age were obtained from the baseline questionnaire for all 1,153 women (Fig. 1). The remaining 11,946 baseline respondents without lumbar MRI scans were used as a reference group.

Between January 1980 and December 2016, 299 (25.9%) of the 1,153 women in the MRI study sample underwent TKA, while 170 (14.7%) underwent THA. However, four study participants had THA, and another four had TKA before the baseline questionnaire date (between December 1980 and May 1989); these individuals were excluded from the final analysis. Thus, the study sample in both the THA and TKA analyses consisted of 1,149 women. In the reference group of 11,946 women, 1,646 (13.8%) women had TKA, and 1036 (8.7%) women had THA during the same follow-up period.

### Disc degeneration grade

The IDD severity was graded at five lumbar intervertebral levels, from the L1-L2 level to the L5-S1 level, from T2-weighted MRI images using the 5-grade Pfirrmann disc degeneration classification system [26]. The intra- [26] and interobserver [23, 26] reliability of this disc degeneration classification system has been reported to be from substantial to excellent. In this classification, the Grade 1 disc is considered a totally healthy disc without signs of degeneration, and Grade 5 is regarded as end-stage degeneration [26].

### Statistical analysis

All statistical analyses in the present study were conducted with SPSS, version 27.0.1.0. The degeneration grade was distributed into two groups at all five studied intervertebral levels: (1) 1 to 4 (non-severe IDD) and (2) 5 (severe IDD). For each participant, a mean degeneration grade of all five studied lumbar intervertebral discs (L1-L2 to L5-S1) was calculated. This mean degeneration grade was further classified into two groups: (1) less than 4 (non-severe IDD) and (2) 4 to 5 (severe IDD). Degeneration classification was structured as previously described by Salo et al. [27].

Differences between the characteristics of the reference group and MRI subsample group were analyzed using the independent samples T-test for all continuous variables. Prior to each t-test, Levene’s test was performed to assess the equality of variances. For categorical variables, comparisons were conducted using the chi-squared test.

The MRI scans and arthroplasties were performed independently over a non-controlled timespan during the follow-up period. To account for the exposure time to severe degeneration, the Cox regression with a time-dependent covariate was used to calculate hazard ratios (HRs) with 95% confidence intervals (CIs) for THA and TKA. The follow-up extended from baseline until the THA/TKA event, death, or the final day of the year 2016, whichever occurred first. Death and the end of follow-up time were considered to cause censoring. IDD was included as a dichotomous time-dependent covariate categorized as non-severe IDD or severe IDD. The non-severe IDD was used as a reference group. *P* < 0.05 was considered statistically significant. In the Cox regression analyses, the severity of IDD was assumed to be similar 5 years before MRI imaging to harmonize the time-dependent survival. To examine the effect of assumed exposure time on severe degeneration hazard ratios, the same analyses were conducted as sensitivity analyses, using exposure times from 10 years before MRI to the date of MRI at half-year intervals. To control for potential confounding factors, the following variables were used as covariates in the adjusted Cox regression analyses: the age on the baseline questionnaire date, BMI, number of medical conditions, leisure time physical activity (average hours per week), and smoking history in years.

### Ethics statement

The study protocol was approved by the Hospital District of Northern Savo Research Ethics Committee and conformed to the Helsinki Declaration of 1975, as revised in 2000. The OSTPRE study has been approved for using CRHC and FAR register data. Informed written consent was collected from the participants.

## Results

### Characteristics and disc degeneration

The characteristics of the study population are presented in Table 1. The differences in the characteristics between the MRI study sample and the reference group were relatively small.

**Table 1:** The characteristics of the study population

	*N* (%)	Mean	SD	Minimum	Maximum	*N* reference group (%)	Mean (ref. group)	SD (ref. group)	*p*-value
Age on the baseline questionnaire date (years)	1153	52.1	2.8	47.4	57.4	11,946	52.3	2.9	0.003
Age on the MRI date (years)	1153	73.0	4.4	61.3	83.3				
Time from baseline questionnaire to MRI (years)	1153	20.9	3.6	13.4	26.6				
Height (cm)	1153	161.7	5.2	143.0	178.0	11,917	161.1	5.3	< 0.001
Weight (kg)	1153	68.5	10.7	44.0	115.0	11,916	68.4	12.1	0.626
Body mass index (m^2^/kg)	1153	26.2	3.8	16.9	42.8	11,902	26.3	4.5	0.260
Medical Conditions	1153	1.3	1.3	0.0	9.0	11,946	1.2	1.2	< 0.001
Regular leisure-time physical activity									0.635
Yes	594 (52.2)					6050 (51.4)			
No	543 (47.8)					5696 (48.5)			
Regular leisure-time physical activity (average hours/week)	1137	2.6	3.6	0.0	30.0	11,750	2.5	3.4	0.222
Ever-smoker	302 (26.6)					2991 (25.6)			0.455
Average smoking history (years)	1134	3.2	7.7	0.0	35.0	11,031	3.3	7.8	0.772
Disc degeneration grade									
L1-L2 degeneration	1153	3.41	0.65	2	5				
L2-L3 degeneration	1153	3.55	0.66	1	5				
L3-L4 degeneration	1153	3.58	0.63	2	5				
L4-L5 degeneration	1153	3.84	0.64	2	5				
L5-S1 degeneration	1153	3.92	0.81	1	5				
L1-S1 mean degeneration grade	1153	3.66	0.41	2.20	4.80				

Differences in the mean values between the MRI subsample group and the reference group were analyzed using independent samples T-test for continuous variables including age, height, weight, BMI, the total amount of medical conditions, average smoking history in years, and leisure time physical activity (average hours per week). The chi-squared test was used for categorical variables, including ever-smoking (Yes/No) and leisure time physical activity (Yes/No)

The total number of evaluated intervertebral discs for the study population (*N* = 1,153) was 5,765. The disc degeneration distribution is presented in Supplementary Table 1. Most of the discs (97.9%) were within the higher degeneration groups 3 to 5. The severity of IDD was more severe at the two lowest (L4-L5 and L5-S1) studied vertebral levels (Table 1 and Supplementary Table 1).

### IDD and arthroplasty hazard

A higher hazard for THA was observed in women with severe IDD at the L1-L2 (HR 2.66, 95% CI 1.48–4.80), L2-L3 (HR 1.97, 95% CI 1.15–3.38), and L5-S1 (HR 1.69, 95% CI 1.16–2.46) levels. Severe mean disc degeneration of all five intervertebral levels of the lumbar spine (L1-S1) was also associated with a higher hazard for THA (HR 2.19, 95% CI 1.53–3.15) (Table 2). The adjustment for potential confounders lowered the HRs for THA at most intervertebral levels, except the L3-L4 level. However, the changes were minor, and the statistical significance of the results was not altered (Table 2). Severe mean disc degeneration of all five intervertebral levels of the lumbar spine (L1-S1) was associated with an elevated hazard for TKA (HR 1.50, 95% CI 1.11–2.02). However, in the adjusted model, the result did not reach statistical significance (HR 1.34, 95% CI 0.98–1.84).

**Table 2:** Hazard ratios for total hip arthroplasty (THA) and total knee arthroplasty (TKA) from Cox regression

Vertebral level	HR	95% CI	*p*-value	Adjusted HR	Adjusted 95% CI	Adjusted *p*-value
	**THA**					
L1-L2	2.66	1.48–4.80	0.001*	2.55	1.40–4.64	0.002*
L2-L3	1.97	1.15–3.38	0.013*	1.80	1.02–3.15	0.042*
L3-L4	1.70	0.91–3.18	0.095	1.75	0.93–3.27	0.082
L4-L5	1.44	0.88–2.35	0.146	1.43	0.86–2.36	0.169
L5-S1	1.69	1.16–2.46	0.006*	1.61	1.10–2.36	0.015*
L1-S1 mean degeneration grade	2.19	1.53–3.15	< 0.001**	2.09	1.44–3.04	< 0.001**
	**TKA**					
L1-L2	1.13	0.57–2.25	0.725	1.07	0.54–2.11	0.851
L2-L3	1.56	0.98–2.47	0.058	1.44	0.90–2.32	0.132
L3-L4	1.06	0.60–1.88	0.835	0.99	0.55–1.78	0.962
L4-L5	1.14	0.75–1.74	0.529	1.21	0.79–1.87	0.377
L5-S1	1.07	0.77–1.49	0.677	0.94	0.67–1.33	0.736
L1-S1 mean degeneration grade	1.50	1.11–2.02	0.008*	1.34	0.98–1.84	0.063

The table represents results from the Cox regression model with a time-dependent covariate. Hazard ratios (HRs) for THA and TKA for the severe degeneration group are presented from the adjusted and unadjusted models. Non-severe degeneration was used as a reference group. Age on the baseline questionnaire date (years), BMI, the total number of medical conditions, leisure time physical activity (average hours per week), and smoking history in years were used as covariates in the adjusted model. The hazard ratios of the covariates are presented in Table 3

*p < 0.05, **p < 0.001

### Confounding factors and other findings

BMI increased the hazard for TKA at all five vertebral levels and when the L1-S1 mean degeneration grade was studied. BMI also increased the hazard for THA, but the effect was not as strong as for the TKA (Table 3).

**Table 3:** Hazard ratios of the covariates in the adjusted Cox regression model

Vertebral level	Covariate	HR (THA)	95% CI (THA)	*p*-value (THA)	HR (TKA)	95% CI (TKA)	*p*-value (TKA)
L1-L2							
	Age on the baseline questionnaire date (years)	1.05	1.00–1.10	0.071	1.02	0.98–1.06	0.375
	BMI	1.04	1.00–1.07	0.061	1.11	1.08–1.14	< 0.001*
	Number of medical conditions	0.97	0.87–1.08	0.589	0.97	0.89–1.06	0.540
	Leisure time physical activity (average hours per week)	1.01	0.98–1.05	0.493	1.02	0.99–1.05	0.197
	Smoking history in years	1.00	0.98–1.02	0.989	0.99	0.97–1.01	0.190
L2-L3							
	Age on the baseline questionnaire date (years)	1.05	0.99–1.10	0.080	1.02	0.98–1.06	0.397
	BMI	1.04	1.00–1.08	0.041*	1.11	1.08–1.14	< 0.001**
	Number of medical conditions	0.97	0.86–1.09	0.597	0.97	0.89–1.06	0.493
	Leisure time physical activity (average hours per week)	1.01	0.98–1.05	0.438	1.02	0.99–1.05	0.186
	Smoking history in years	1.00	0.98–1.02	0.975	0.99	0.97–1.01	0.195
L3-L4							
	Age on the baseline questionnaire date (years)	1.05	1.00–1.10	0.072	1.02	0.98–1.06	0.375
	BMI	1.04	1.00–1.07	0.041*	1.11	1.08–1.14	< 0.001**
	Number of medical conditions	0.98	0.87–1.09	0.671	0.97	0.89–1.06	0.541
	Leisure time physical activity (average hours per week)	1.01	0.98–1.05	0.523	1.02	0.99–1.05	0.198
	Smoking history in years	1.00	0.98–1.02	0.947	0.99	0.97–1.01	0.192
L4-L5							
	Age on the baseline questionnaire date (years)	1.05	0.99–1.10	0.088	1.02	0.98–1.06	0.393
	BMI	1.04	1.00–1.08	0.039*	1.11	1.08–1.14	< 0.001**
	Number of medical conditions	0.97	0.87–1.09	0.652	0.97	0.89–1.06	0.545
	Leisure time physical activity (average hours per week)	1.01	0.97–1.05	0.553	1.02	0.99–1.05	0.216
	Smoking history in years	1.00	0.98–1.02	0.986	0.99	0.97–1.01	0.180
L5-S1							
	Age on the baseline questionnaire date (years)	1.04	0.99–1.10	0.115	1.02	0.98–1.06	0.366
	BMI	1.04	1.00–1.07	0.049*	1.11	1.08–1.14	< 0.001**
	Number of medical conditions	0.97	0.87–1.09	0.644	0.97	0.89–1.06	0.544
	Leisure time physical activity (average hours per week)	1.02	0.98–1.05	0.431	1.02	0.99–1.05	0.198
	Smoking history in years	1.00	0.98–1.02	0.930	0.99	0.97–1.01	0.190
L1-S1							
	Age on the baseline questionnaire date (years)	1.04	0.99–1.09	0.135	1.02	0.97–1.06	0.447
	BMI	1.03	1.00–1.07	0.088	1.11	1.08–1.14	< 0.001**
	Number of medical conditions	0.98	0.87–1.10	0.702	0.97	0.89–1.07	0.575
	Leisure time physical activity (average hours per week)	1.01	0.97–1.05	0.531	1.02	0.99–1.05	0.209
	Smoking history in years	1.00	0.98–1.02	0.977	0.99	0.97–1.01	0.177

Hazard ratios (HRs) for covariates in the adjusted Cox regression model are presented in the table from total hip arthroplasty (THA) analysis and total knee arthroplasty (TKA) analysis. Hazard ratios for THA and TKA are presented in Table 2

* *p* < 0.05, ** *p* < 0.001

In the Cox regression model, the degeneration grade was assumed to be similar 5 years before MRI. Supplementary Table 2 A–F presents how this assumed exposure time affects hazard ratios for THA at each vertebral level when the assumed time varies from − 10 to 0 years before the MRI. Supplementary Table 3 A–F presents the same information according to the TKA analysis.

The proportion of severe and non-severe degeneration at different vertebral levels is presented in Supplementary Table 4. The distribution of indications or diagnoses related to lumbar MRI is presented in Supplementary Table 5. The largest diagnosis group was spinal stenosis, comprising 46.1% of all MRI scans. The indications of performed arthroplasties are presented in Supplementary Table 6. The majority of TKAs (94.0%) and THAs (82.9%) were performed due to primary OA.

## Discussion

The present population-based cohort study investigated the association between lumbar IDD and clinically evident hip and knee OA, indicated by arthroplasties. It was found that women with severe disc degeneration had a higher hazard (HR 1.44–2.66 depending on vertebral level) for THA. The association between severe disc degeneration and a higher hazard for THA was observed at all vertebral levels. This result was statistically significant at the L1-L2, L2-L3, and L5-S1 intervertebral levels and also when the mean degeneration grade of all five lumbar intervertebral levels (from L1-L2 to L5-S1 level) was used in the analysis. Women with severe degeneration also seemed to have a slightly elevated hazard (HR 1.06–1.56 depending on vertebral level) for TKA. However, these results were statistically significant only in the mean degeneration grade of all five lumbar intervertebral level analyses. In the adjusted model, the statistical significance of this result was lost. BMI was the only statistically significant covariate in the analyses, and higher BMI increased the hazard for both THA and TKA; however, the effect was more substantial for TKA.

LBP has been found to be a frequent musculoskeletal comorbidity among hip OA patients, and 40.8–60.4% of patients with severe hip OA have been reported to have concurrent LBP [28–30]. The prevalence of back pain has been found to be from 54.6 to 57.4% among patients with knee OA [31, 32]. However, the prevalence of moderate to severe back pain preoperatively seems to be higher among THA patients than among TKA patients (28.8% vs. 16.1%) [28]. It may be challenging to distinguish whether the lower leg pain originates from a spinal nerve block or hip OA [33]. Concurrent hip OA and degenerative lumbar spinal stenosis are not rare in the elderly population; this combination, called hip-spine syndrome, was first described by Offierski and MacNab in the early 1980s [34]. Hip-spine syndrome was clinically validated for the first time 24 years later, and it was also found that both LBP and spinal function were improved following THA [35]. THA can relieve LBP by relieving hip pain and restoring hip function in patients with both lumbar and hip degenerative disease, possibly avoiding further spinal surgery [36]. THA seems to improve lumbar flexibility and, interestingly, also intervertebral disc height [37]. However, there appears to be a significant increase in the risk of complications after THA in patients with lumbar spine disease [38]. In addition, TKA outcomes may be impaired by the coexistence of lumbar IDD [39].

Previous studies indicate that spinopelvic alignment is associated with both IDD and OA. Pelvic tilt (PT) and sacral slope (SS) are considered parameters for sagittal pelvic orientation, while PI is considered to define sagittal pelvic morphology [40]. Van Erp et al. found in an observational cohort study that high PI is a risk factor for the development of spondylolisthesis and knee OA [21]. Subsequently, an extensive systematic review found similar results and concluded that high PI is a risk factor for the development of spondylolisthesis and knee OA [41]. Furthermore, low PI was found to be a risk factor for DDD [21]. A meta-analysis concluded that degenerative spondylolisthesis is connected to significantly higher PI [42]. Pelvic incidence-lumbar lordosis (PI-LL) mismatch is used to evaluate clinical outcomes in patients with sagittal malalignment. PI-LL mismatch was found to be associated with IDD [43]. The relationship between PI and hip OA seems to be much more unclear. A review study summarized that the evidence on the association between PI and hip OA remained inconclusive [44]. Later, it was found that individuals with low PI had a higher incidence of hip OA compared to those with normal or high PI, and it was concluded that low PI may be associated with the development of hip OA [21]. However, a narrative review recently investigated the relationship between PI and hip disorders, and it was summarized that the association between PI and hip disorders still remains controversial [45]. It was discussed that the multifactorial nature of hip OA and the wide range of PIs in Hip OA make interpreting the results difficult. Additionally, advanced hip OA may theoretically increase PI by causing cranial displacement of the hip due to femoral head destruction and subluxation, as well as altering sacroiliac joint angulation through accelerated joint degeneration. This dual effect could contribute to changes in pelvic morphology over time [45]. In the present study, these spinal parameters were not evaluated. However, it has been found that supine MRI underestimates lumbopelvic sagittal alignment parameters compared to standing radiographs. Standing lumbar radiographs are better for assessing these parameters [46]. Our IDD data was based on lumbar MRIs performed due to clinical indications. Concurrent standing lumbar radiographs taken simultaneously with lumbar MRI were not available in this study. However, future studies should combine data from lumbar radiographs, MRI, and arthroplasties to provide a comprehensive understanding of the relationship between IDD and OA.

Early lumbar DDD has been found to be twice as common as hip OA changes in the early 20s age range [11]. Based on a cadaveric study, it has been suggested that lumbar degeneration precedes hip degeneration and may be a causative factor for hip OA [11]. Higher hip and knee OA prevalence was found in individuals with radiographic signs of spinal degeneration [14]. When the association between knee OA and degenerative changes in the lumbar spine was examined, it was found that knee OA was associated with osteophytes and facet joint OA but not with disc space narrowing [16]. However, no associations were found between hip OA and spinal degeneration signs [16]. The present study combined two major components of IDD, including disc space narrowing and signal intensity decrease, by using an MRI-based classification system introduced by Pfirrmann et al. [26]. It was found that women who had severe IDD in the lumbar spine had a higher hazard for THA. A weak association between severe IDD and increased hazard for TKA was found. However, this association was statistically significant in only the L1-S1 mean degeneration grade analysis, and in the adjusted model, statistical significance was lost. The results of the present study support the association between severe IDD and severe hip OA. According to the results, the association between knee OA and IDD seems to be weaker than that between hip OA and IDD.

IDD and OA share several mutual risk factors. Aging is known to be one of the most important risk factors for IDD [47, 48] but also OA [49]. Being overweight is strongly associated with an increased risk of IDD in the lumbar spine [50]. Higher BMI has also been associated with an increased risk for hip and knee OA [51]. Obesity has been suggested to be the main modifiable risk factor for knee OA [52]. The results of the present study support the association between higher BMI and OA, as BMI increased the hazard for both THA and TKA. However, the effect seemed to be stronger for the TKA hazard. The association between higher BMI and knee OA seems stronger than between higher BMI and hip OA. Therefore, a higher BMI might be a more significant risk factor for knee OA than hip OA. However, the present study’s design allows no conclusions about the causality between a higher BMI and OA. In addition, the smoking history in years seemed not to increase the THA or TKA hazard in the present study. Some women in the study population had more than one TKA or THA; some were revisions, and some were the first total arthroplasties of the other side joint. Factors associated with revisions or more than one arthroplasty should be investigated in further studies.

The MRI scans and arthroplasties were independently performed over a non-controlled timespan during the follow-up. In the Cox regression analysis, the degeneration severity was assumed to be similar 5 years before MRI. The assumed exposure time for severe degeneration affects the HRs for arthroplasty. However, Cox regression analyses were also performed, with the degeneration grade assumed to be similar from 10 to 0 years before MRI (Supplementary tables 2 A–F and 3 A–F). While looking at the L1-S1 mean degeneration grade analysis, it was found that hazard ratios for TKA increased with longer assumed exposure time for severe degeneration. However, the effect of a longer assumed exposure time was controversial for THA hazard in the L1-S1 mean degeneration analysis. The THA hazard ratio decreased with a higher assumed exposure time for severe degeneration. However, with any assumed exposure time for degeneration, the association between severe IDD and increased THA hazard seemed to be stronger than that between severe IDD and increased TKA hazard.

Overall, the proportion of severe degeneration was highest in the lower lumbar spine (L4-L5 and L5-S1). However, women with severe degeneration in the upper intervertebral levels (especially L1-L2) had a higher hazard for THA than women with severe degeneration in the lower spine. This may be related to higher overall degeneration since the L1-S1 mean degeneration grade was 4.31 in women with severe degeneration at the L1-L2 level (*N* = 59) and 3.95 in women with severe degeneration at the L5-S1 level (*N* = 296). (data not shown).

The strengths of the present study include a large population-based study sample, including an entire age cohort, resulting in a relatively large sample size and a representative study population. To the best of our knowledge, the present study is the first to investigate the association between MRI-evaluated severe IDD and the hazard of THA and TKA. Additionally, it has the largest sample size of the published studies thus far that investigated the association between hip and knee OA and IDD evaluated from MRI images. A major strength of the study is that it combined data from a large cohort and clinical patient data. The follow-up time from the baseline questionnaire in 1989 until the end of 2016 was over 26 years. The disc degeneration grade was evaluated blinded to the THA and TKA data prior to analyses.

Several limitations are associated with the methodology and framework of the present study. First, the final study population of 1,149 women represented only a small proportion of the original OSTPRE study cohort, and selection bias is possible in the study. Furthermore, the study population consisted only of women, and the findings could have been different in a male population. However, previous studies have found evidence of the association between OA and IDD in study populations containing both females and males. Based on a cadaveric study (87.6% male, 12.4% female), Bajwa et al. suggested that lumbar degeneration precedes hip degeneration and may be a causative factor for hip OA [11]. Another study, with a mixed-sex population (55.1% female, 44.9% male), found higher osteoarthritis prevalence in individuals with radiographic signs of spinal degeneration [14]. Furthermore, a study with a mixed-sex population (58.9% female, 41.1% male) found that the OA burden was independently associated with the severity of IDD [18]. Though the results of the present study could have been different in a male population, based on the previous studies, it is likely that the results would have been parallel in a male population as well. All MRI scans were performed due to clinical indications of lumbar MRI, which may introduce some selection bias. Hence, individuals with clinical lower back problems were clearly overrepresented in the present study sample. The severity of IDD would have likely been less severe for a random sample of the OSTPRE study population, which may have also affected the study results. While physicians generally adhere to established guidelines for lumbar MRI, the decision to perform these scans may vary among practitioners and depend on individual patient-related factors. MRI scans and arthroplasties were performed independently at different time points. However, the Cox regression model with a time-dependent covariate was used in the analysis to account for this. In addition, the present study’s design allows no conclusions about the causality between more severe IDD and OA. Finally, the average participant age, height, and total number of medical conditions differed slightly between the MRI subsample and the whole OSTPRE study cohort reference group. Although these differences were statistically significant, they were still relatively small. It was concluded that the MRI group was a rather representative subsample of the original OSTPRE study cohort.

## Conclusion

In conclusion, the present study supports the association between disc degeneration in the lumbar spine and osteoarthritis of the hip in postmenopausal women. Women with severe IDD had a higher hazard for THA. A parallel but weak association was obtained between IDD and TKA hazard. However, this result was significant only when the mean degeneration grade of all five lumbar intervertebral levels was used in the analysis, and statistical significance was lost in the adjusted model with potential confounders. Higher BMI increased the hazard for both THA and TKA in women with severe IDD in the lumbar spine. This effect seemed to be stronger for the TKA hazard. The present study substantially increases the knowledge of the association between IDD and OA. However, the causality between IDD and OA should be more thoroughly studied in the future.

## Electronic supplementary material

Below is the link to the electronic supplementary material.

Supplementary Material 1

Supplementary Material 2

Supplementary Material 3

Supplementary Material 4

Supplementary Material 5

Supplementary Material 6

## Data Availability

The data that support the findings of this study are not openly available due to reasons of sensitivity and are available from the corresponding author upon reasonable request.
