# Supplementary material for: Association between severe lumbar disc degeneration and end-stage hip or knee osteoarthritis requiring joint replacement surgery: a population-based cohort study with a 26-year follow-up
Source: Arch Orthop Trauma Surg. 2025 May 12;145(1):288. doi: 10.1007/s00402-025-05908-7 (PMC12069494; doi:10.1007/s00402-025-05908-7)
Supplement: Supplementary file 1 — Supplementary Material 1 [file 402_2025_5908_MOESM1_ESM.docx]

**Supplementary Table 1. Distribution of disc degeneration grades (N=1,153)**

| Pfirrmann grade | L1-L2 | L2-L3 | L3-L4 | L4-L5 | L5-S1 | Total |
| --- | --- | --- | --- | --- | --- | --- |
| 1 | 0 | 1 (0.1%) | 0 | 0 | 2 (0.2%) | 3 (0.1%) |
| 2 | 45 (3.9%) | 16 (1.4%) | 11 (1.0%) | 6 (0.5%) | 39 (3.4%) | 117 (2.0%) |
| 3 | 655 (56.8%) | 572 (49.6%) | 543 (47.1%) | 320 (27.8%) | 300 (26.0%) | 2390 (41.5%) |
| 4 | 394 (34.2%) | 474 (41.1%) | 519 (45.0%) | 676 (58.6%) | 516 (44.8%) | 2579 (44.7%) |
| 5 | 59 (5.1%) | 90 (7.8%) | 80 (6.9%) | 151 (13.1%) | 296 (25.7%) | 676 (11.7%) |
