# Supplementary material for: Association between severe lumbar disc degeneration and end-stage hip or knee osteoarthritis requiring joint replacement surgery: a population-based cohort study with a 26-year follow-up
Source: Arch Orthop Trauma Surg. 2025 May 12;145(1):288. doi: 10.1007/s00402-025-05908-7 (PMC12069494; doi:10.1007/s00402-025-05908-7)
Supplement: Supplementary file 2 — Supplementary Material 2 [file 402_2025_5908_MOESM2_ESM.docx]

**Supplementary Table 2A-F and supplementary figures. Hazard ratios for THA in severe disc degeneration groups from the Cox regression model with time-dependent covariate. Degeneration grade assumed to be similar -10 to 0 years before MRI**

**Supplementary Table 2A: L1-L2 severe degeneration and THA hazard ratio**

| Years from MRI | Hazard ratio (HR) | 95% CI Lower | 95% CI Upper | p-value |
| --- | --- | --- | --- | --- |
| -10.00 | 2.34 | 1.36 | 4.02 | 0.002 |
| -9.50 | 2.39 | 1.39 | 4.12 | 0.002 |
| -9.00 | 2.24 | 1.29 | 3.91 | 0.004 |
| -8.50 | 2.11 | 1.19 | 3.74 | 0.011 |
| -8.00 | 2.17 | 1.22 | 3.86 | 0.008 |
| -7.50 | 2.25 | 1.26 | 4.00 | 0.006 |
| -7.00 | 2.32 | 1.30 | 4.14 | 0.005 |
| -6.50 | 2.39 | 1.34 | 4.28 | 0.003 |
| -6.00 | 2.47 | 1.38 | 4.44 | 0.002 |
| -5.50 | 2.56 | 1.43 | 4.60 | 0.002 |
| -5.00 | 2.66 | 1.48 | 4.80 | 0.001 |
| -4.50 | 2.51 | 1.37 | 4.59 | 0.003 |
| -4.00 | 2.36 | 1.26 | 4.39 | 0.007 |
| -3.50 | 2.46 | 1.32 | 4.61 | 0.005 |
| -3.00 | 2.32 | 1.21 | 4.43 | 0.011 |
| -2.50 | 2.45 | 1.28 | 4.69 | 0.007 |
| -2.00 | 2.64 | 1.37 | 5.09 | 0.004 |
| -1.50 | 2.52 | 1.27 | 5.02 | 0.008 |
| -1.00 | 2.72 | 1.36 | 5.43 | 0.005 |
| -0.50 | 3.02 | 1.64 | 5.55 | <0.001 |
| 0.00 | 3.09 | 1.64 | 5.82 | 0.001 |

**Supplementary Table 2B: L2-L3 severe degeneration and THA hazard ratio**

| Years from MRI | Hazard ratio (HR) | 95% CI Lower | 95% CI Upper | p-value |
| --- | --- | --- | --- | --- |
| -10.00 | 1.81 | 1.10 | 2.96 | 0.020 |
| -9.50 | 1.84 | 1.12 | 3.03 | 0.016 |
| -9.00 | 1.76 | 1.06 | 2.93 | 0.029 |
| -8.50 | 1.81 | 1.09 | 3.01 | 0.022 |
| -8.00 | 1.87 | 1.12 | 3.11 | 0.017 |
| -7.50 | 1.92 | 1.15 | 3.21 | 0.012 |
| -7.00 | 1.98 | 1.19 | 3.31 | 0.009 |
| -6.50 | 1.91 | 1.13 | 3.22 | 0.015 |
| -6.00 | 1.98 | 1.17 | 3.34 | 0.011 |
| -5.50 | 1.89 | 1.11 | 3.23 | 0.020 |
| -5.00 | 1.97 | 1.15 | 3.38 | 0.013 |
| -4.50 | 2.05 | 1.20 | 3.52 | 0.009 |
| -4.00 | 2.14 | 1.24 | 3.68 | 0.006 |
| -3.50 | 2.25 | 1.30 | 3.87 | 0.004 |
| -3.00 | 2.18 | 1.25 | 3.81 | 0.006 |
| -2.50 | 2.31 | 1.32 | 4.06 | 0.003 |
| -2.00 | 2.51 | 1.42 | 4.43 | 0.002 |
| -1.50 | 2.72 | 1.53 | 4.83 | 0.001 |
| -1.00 | 2.96 | 1.66 | 5.28 | <0.001 |
| -0.50 | 3.02 | 1.64 | 5.55 | <0.001 |
| 0.00 | 3.09 | 1.64 | 5.82 | 0.001 |

**Supplementary Table 2C: L3-L4 severe degeneration and THA hazard ratio**

| Years from MRI | Hazard ratio (HR) | 95% CI Lower | 95% CI Upper | p-value |
| --- | --- | --- | --- | --- |
| -10.00 | 1.64 | 0.95 | 2.85 | 0.078 |
| -9.50 | 1.68 | 0.96 | 2.92 | 0.067 |
| -9.00 | 1.71 | 0.98 | 2.99 | 0.058 |
| -8.50 | 1.76 | 1.01 | 3.08 | 0.047 |
| -8.00 | 1.81 | 1.03 | 3.16 | 0.039 |
| -7.50 | 1.87 | 1.06 | 3.28 | 0.030 |
| -7.00 | 1.93 | 1.09 | 3.39 | 0.024 |
| -6.50 | 1.99 | 1.13 | 3.52 | 0.018 |
| -6.00 | 1.90 | 1.06 | 3.41 | 0.032 |
| -5.50 | 1.64 | 0.88 | 3.06 | 0.118 |
| -5.00 | 1.70 | 0.91 | 3.18 | 0.095 |
| -4.50 | 1.77 | 0.94 | 3.31 | 0.075 |
| -4.00 | 1.85 | 0.98 | 3.49 | 0.056 |
| -3.50 | 1.75 | 0.91 | 3.37 | 0.093 |
| -3.00 | 1.65 | 0.83 | 3.26 | 0.153 |
| -2.50 | 1.76 | 0.88 | 3.50 | 0.110 |
| -2.00 | 1.88 | 0.94 | 3.78 | 0.075 |
| -1.50 | 2.04 | 1.01 | 4.11 | 0.046 |
| -1.00 | 1.96 | 0.94 | 4.10 | 0.075 |
| -0.50 | 1.88 | 0.85 | 4.15 | 0.119 |
| 0.00 | 1.76 | 0.76 | 4.11 | 0.190 |

**Supplementary Table 2D: L4-L5 severe degeneration and THA hazard ratio**

| Years from MRI | Hazard ratio (HR) | 95% CI Lower | 95% CI Upper | p-value |
| --- | --- | --- | --- | --- |
| -10.00 | 1.21 | 0.76 | 1.90 | 0.422 |
| -9.50 | 1.23 | 0.78 | 1.95 | 0.371 |
| -9.00 | 1.19 | 0.75 | 1.90 | 0.468 |
| -8.50 | 1.15 | 0.71 | 1.85 | 0.570 |
| -8.00 | 1.18 | 0.73 | 1.90 | 0.504 |
| -7.50 | 1.21 | 0.75 | 1.96 | 0.433 |
| -7.00 | 1.25 | 0.77 | 2.01 | 0.370 |
| -6.50 | 1.29 | 0.79 | 2.08 | 0.308 |
| -6.00 | 1.33 | 0.82 | 2.16 | 0.246 |
| -5.50 | 1.38 | 0.85 | 2.25 | 0.193 |
| -5.00 | 1.44 | 0.88 | 2.35 | 0.146 |
| -4.50 | 1.50 | 0.92 | 2.46 | 0.108 |
| -4.00 | 1.58 | 0.96 | 2.59 | 0.073 |
| -3.50 | 1.45 | 0.86 | 2.43 | 0.162 |
| -3.00 | 1.42 | 0.84 | 2.42 | 0.193 |
| -2.50 | 1.40 | 0.81 | 2.41 | 0.228 |
| -2.00 | 1.49 | 0.86 | 2.58 | 0.152 |
| -1.50 | 1.36 | 0.77 | 2.43 | 0.293 |
| -1.00 | 1.34 | 0.73 | 2.43 | 0.343 |
| -0.50 | 1.19 | 0.62 | 2.26 | 0.606 |
| 0.00 | 1.31 | 0.68 | 2.51 | 0.421 |

**Supplementary Table 2E: L5-S1 severe degeneration and THA hazard ratio**

| Years from MRI | Hazard ratio (HR) | 95% CI Lower | 95% CI Upper | p-value |
| --- | --- | --- | --- | --- |
| -10.00 | 1.46 | 1.03 | 2.07 | 0.034 |
| -9.50 | 1.45 | 1.02 | 2.06 | 0.037 |
| -9.00 | 1.44 | 1.02 | 2.05 | 0.041 |
| -8.50 | 1.39 | 0.98 | 1.99 | 0.067 |
| -8.00 | 1.44 | 1.01 | 2.06 | 0.047 |
| -7.50 | 1.49 | 1.04 | 2.13 | 0.030 |
| -7.00 | 1.49 | 1.04 | 2.14 | 0.031 |
| -6.50 | 1.55 | 1.07 | 2.23 | 0.019 |
| -6.00 | 1.61 | 1.11 | 2.32 | 0.011 |
| -5.50 | 1.68 | 1.16 | 2.43 | 0.006 |
| -5.00 | 1.69 | 1.16 | 2.46 | 0.006 |
| -4.50 | 1.65 | 1.12 | 2.41 | 0.011 |
| -4.00 | 1.61 | 1.09 | 2.37 | 0.017 |
| -3.50 | 1.57 | 1.06 | 2.33 | 0.026 |
| -3.00 | 1.68 | 1.12 | 2.50 | 0.012 |
| -2.50 | 1.65 | 1.09 | 2.48 | 0.017 |
| -2.00 | 1.63 | 1.07 | 2.49 | 0.023 |
| -1.50 | 1.69 | 1.10 | 2.60 | 0.017 |
| -1.00 | 1.76 | 1.13 | 2.74 | 0.012 |
| -0.50 | 1.74 | 1.10 | 2.76 | 0.017 |
| 0.00 | 1.84 | 1.15 | 2.95 | 0.011 |

**Supplementary Table 2F: L1-S1 severe mean degeneration and THA hazard ratio**

| Years from MRI | Hazard ratio (HR) | 95% CI Lower | 95% CI Upper | p-value |
| --- | --- | --- | --- | --- |
| -10.00 | 1.96 | 1.41 | 2.72 | <0.001 |
| -9.50 | 2.02 | 1.45 | 2.81 | <0.001 |
| -9.00 | 1.96 | 1.40 | 2.74 | <0.001 |
| -8.50 | 2.03 | 1.45 | 2.85 | <0.001 |
| -8.00 | 2.04 | 1.45 | 2.87 | <0.001 |
| -7.50 | 2.12 | 1.50 | 3.00 | <0.001 |
| -7.00 | 2.08 | 1.47 | 2.94 | <0.001 |
| -6.50 | 2.10 | 1.48 | 2.98 | <0.001 |
| -6.00 | 2.13 | 1.49 | 3.03 | <0.001 |
| -5.50 | 2.09 | 1.46 | 2.98 | <0.001 |
| -5.00 | 2.19 | 1.53 | 3.15 | <0.001 |
| -4.50 | 2.24 | 1.55 | 3.23 | <0.001 |
| -4.00 | 2.22 | 1.53 | 3.22 | <0.001 |
| -3.50 | 2.20 | 1.51 | 3.21 | <0.001 |
| -3.00 | 2.27 | 1.54 | 3.33 | <0.001 |
| -2.50 | 2.26 | 1.53 | 3.35 | <0.001 |
| -2.00 | 2.37 | 1.59 | 3.52 | <0.001 |
| -1.50 | 2.50 | 1.66 | 3.75 | <0.001 |
| -1.00 | 2.53 | 1.67 | 3.82 | <0.001 |
| -0.50 | 2.58 | 1.68 | 3.95 | <0.001 |
| 0.00 | 2.78 | 1.79 | 4.31 | <0.001 |
