# Supplementary material for: Association between severe lumbar disc degeneration and end-stage hip or knee osteoarthritis requiring joint replacement surgery: a population-based cohort study with a 26-year follow-up
Source: Arch Orthop Trauma Surg. 2025 May 12;145(1):288. doi: 10.1007/s00402-025-05908-7 (PMC12069494; doi:10.1007/s00402-025-05908-7)
Supplement: Supplementary file 3 — Supplementary Material 3 [file 402_2025_5908_MOESM3_ESM.docx]

**Supplementary Table 3A-F and supplementary figures. Hazard ratios for TKA in severe disc degeneration groups, from Cox regression model. Degeneration grade assumed to be similar -10 to 0 years before MRI**

**Supplementary Table 3A: L1-L2 severe degeneration and TKA hazard ratio**

| Years from MRI | Hazard ratio (HR) | 95% CI Lower | 95% CI Upper | p-value |
| --- | --- | --- | --- | --- |
| -10.00 | 1.40 | 0.81 | 2.41 | 0.228 |
| -9.50 | 1.43 | 0.83 | 2.48 | 0.197 |
| -9.00 | 1.48 | 0.85 | 2.57 | 0.165 |
| -8.50 | 1.52 | 0.87 | 2.65 | 0.139 |
| -8.00 | 1.45 | 0.82 | 2.58 | 0.199 |
| -7.50 | 1.38 | 0.77 | 2.49 | 0.279 |
| -7.00 | 1.32 | 0.72 | 2.43 | 0.373 |
| -6.50 | 1.37 | 0.74 | 2.52 | 0.318 |
| -6.00 | 1.17 | 0.61 | 2.25 | 0.642 |
| -5.50 | 1.21 | 0.63 | 2.34 | 0.570 |
| -5.00 | 1.13 | 0.57 | 2.25 | 0.725 |
| -4.50 | 1.06 | 0.51 | 2.18 | 0.886 |
| -4.00 | 1.11 | 0.53 | 2.31 | 0.779 |
| -3.50 | 1.17 | 0.56 | 2.45 | 0.672 |
| -3.00 | 0.92 | 0.40 | 2.11 | 0.840 |
| -2.50 | 0.98 | 0.43 | 2.27 | 0.966 |
| -2.00 | 1.05 | 0.45 | 2.46 | 0.903 |
| -1.50 | 0.94 | 0.37 | 2.38 | 0.900 |
| -1.00 | 0.81 | 0.29 | 2.27 | 0.692 |
| -0.50 | 0.89 | 0.32 | 2.50 | 0.824 |
| 0.00 | 0.73 | 0.22 | 2.36 | 0.593 |

**Supplementary Table 3B: L2-L3 severe degeneration and TKA hazard ratio**

| Years from MRI | Hazard ratio (HR) | 95% CI Lower | 95% CI Upper | p-value |
| --- | --- | --- | --- | --- |
| -10.00 | 1.43 | 0.95 | 2.17 | 0.088 |
| -9.50 | 1.40 | 0.92 | 2.14 | 0.114 |
| -9.00 | 1.44 | 0.95 | 2.20 | 0.090 |
| -8.50 | 1.48 | 0.97 | 2.27 | 0.068 |
| -8.00 | 1.53 | 1.00 | 2.34 | 0.051 |
| -7.50 | 1.50 | 0.98 | 2.31 | 0.065 |
| -7.00 | 1.48 | 0.95 | 2.29 | 0.081 |
| -6.50 | 1.54 | 0.99 | 2.39 | 0.057 |
| -6.00 | 1.51 | 0.96 | 2.36 | 0.072 |
| -5.50 | 1.49 | 0.95 | 2.35 | 0.086 |
| -5.00 | 1.56 | 0.98 | 2.47 | 0.058 |
| -4.50 | 1.65 | 1.04 | 2.62 | 0.035 |
| -4.00 | 1.55 | 0.95 | 2.51 | 0.078 |
| -3.50 | 1.64 | 1.01 | 2.68 | 0.047 |
| -3.00 | 1.64 | 0.99 | 2.71 | 0.054 |
| -2.50 | 1.65 | 0.98 | 2.76 | 0.060 |
| -2.00 | 1.64 | 0.96 | 2.81 | 0.069 |
| -1.50 | 1.65 | 0.95 | 2.88 | 0.076 |
| -1.00 | 1.67 | 0.94 | 2.97 | 0.082 |
| -0.50 | 1.68 | 0.92 | 3.08 | 0.091 |
| 0.00 | 1.68 | 0.90 | 3.15 | 0.106 |

**Supplementary Table 3C: L3-L4 severe degeneration and TKA hazard ratio**

| Years from MRI | Hazard ratio (HR) | 95% CI Lower | 95% CI Upper | p-value |
| --- | --- | --- | --- | --- |
| -10.00 | 1.12 | 0.69 | 1.83 | 0.640 |
| -9.50 | 1.15 | 0.70 | 1.88 | 0.577 |
| -9.00 | 1.10 | 0.67 | 1.82 | 0.699 |
| -8.50 | 1.13 | 0.69 | 1.87 | 0.629 |
| -8.00 | 1.16 | 0.70 | 1.93 | 0.555 |
| -7.50 | 1.12 | 0.67 | 1.88 | 0.664 |
| -7.00 | 1.16 | 0.69 | 1.95 | 0.579 |
| -6.50 | 1.20 | 0.71 | 2.01 | 0.501 |
| -6.00 | 1.15 | 0.67 | 1.97 | 0.607 |
| -5.50 | 1.11 | 0.64 | 1.92 | 0.713 |
| -5.00 | 1.06 | 0.60 | 1.88 | 0.835 |
| -4.50 | 1.12 | 0.63 | 1.98 | 0.707 |
| -4.00 | 0.97 | 0.52 | 1.79 | 0.909 |
| -3.50 | 1.02 | 0.55 | 1.89 | 0.962 |
| -3.00 | 0.97 | 0.51 | 1.84 | 0.914 |
| -2.50 | 1.03 | 0.54 | 1.98 | 0.925 |
| -2.00 | 1.10 | 0.57 | 2.12 | 0.769 |
| -1.50 | 1.05 | 0.53 | 2.09 | 0.884 |
| -1.00 | 0.99 | 0.48 | 2.05 | 0.975 |
| -0.50 | 1.08 | 0.52 | 2.25 | 0.836 |
| 0.00 | 1.19 | 0.57 | 2.49 | 0.642 |

**Supplementary Table 3D: L4-L5 severe degeneration and TKA hazard ratio**

| Years from MRI | Hazard ratio (HR) | 95% CI Lower | 95% CI Upper | p-value |
| --- | --- | --- | --- | --- |
| -10.00 | 1.43 | 1.02 | 2.01 | 0.040 |
| -9.50 | 1.34 | 0.95 | 1.90 | 0.099 |
| -9.00 | 1.34 | 0.94 | 1.90 | 0.107 |
| -8.50 | 1.38 | 0.97 | 1.97 | 0.078 |
| -8.00 | 1.42 | 0.99 | 2.03 | 0.055 |
| -7.50 | 1.47 | 1.02 | 2.11 | 0.037 |
| -7.00 | 1.48 | 1.02 | 2.13 | 0.038 |
| -6.50 | 1.44 | 0.99 | 2.09 | 0.059 |
| -6.00 | 1.44 | 0.99 | 2.12 | 0.060 |
| -5.50 | 1.20 | 0.80 | 1.80 | 0.390 |
| -5.00 | 1.14 | 0.75 | 1.74 | 0.529 |
| -4.50 | 1.15 | 0.75 | 1.76 | 0.536 |
| -4.00 | 1.09 | 0.70 | 1.71 | 0.695 |
| -3.50 | 1.09 | 0.69 | 1.73 | 0.701 |
| -3.00 | 0.98 | 0.60 | 1.60 | 0.938 |
| -2.50 | 0.98 | 0.59 | 1.62 | 0.944 |
| -2.00 | 0.99 | 0.59 | 1.66 | 0.954 |
| -1.50 | 0.92 | 0.53 | 1.59 | 0.756 |
| -1.00 | 0.92 | 0.52 | 1.62 | 0.763 |
| -0.50 | 1.00 | 0.56 | 1.79 | 0.989 |
| 0.00 | 1.01 | 0.55 | 1.84 | 0.976 |

**Supplementary Table 3E: L5-S1 severe degeneration and TKA hazard ratio**

| Years from MRI | Hazard ratio (HR) | 95% CI Lower | 95% CI Upper | p-value |
| --- | --- | --- | --- | --- |
| -10.00 | 1.20 | 0.91 | 1.60 | 0.203 |
| -9.50 | 1.21 | 0.91 | 1.61 | 0.191 |
| -9.00 | 1.20 | 0.90 | 1.60 | 0.223 |
| -8.50 | 1.24 | 0.92 | 1.66 | 0.157 |
| -8.00 | 1.20 | 0.89 | 1.61 | 0.232 |
| -7.50 | 1.21 | 0.90 | 1.64 | 0.208 |
| -7.00 | 1.10 | 0.81 | 1.49 | 0.551 |
| -6.50 | 1.14 | 0.84 | 1.56 | 0.397 |
| -6.00 | 1.16 | 0.85 | 1.59 | 0.345 |
| -5.50 | 1.08 | 0.78 | 1.49 | 0.650 |
| -5.00 | 1.07 | 0.77 | 1.49 | 0.677 |
| -4.50 | 0.98 | 0.70 | 1.38 | 0.922 |
| -4.00 | 0.98 | 0.69 | 1.39 | 0.899 |
| -3.50 | 1.01 | 0.71 | 1.44 | 0.964 |
| -3.00 | 0.98 | 0.68 | 1.41 | 0.895 |
| -2.50 | 0.87 | 0.59 | 1.29 | 0.492 |
| -2.00 | 0.90 | 0.60 | 1.35 | 0.609 |
| -1.50 | 0.94 | 0.62 | 1.42 | 0.758 |
| -1.00 | 0.94 | 0.62 | 1.44 | 0.780 |
| -0.50 | 0.94 | 0.61 | 1.47 | 0.795 |
| 0.00 | 0.94 | 0.60 | 1.49 | 0.804 |

**Supplementary Table 3F: L1-S1 severe mean degeneration and TKA hazard ratio**

| Years from MRI | Hazard ratio (HR) | 95% CI Lower | 95% CI Upper | p-value |
| --- | --- | --- | --- | --- |
| -10.00 | 1.62 | 1.25 | 2.12 | 0.000 |
| -9.50 | 1.59 | 1.22 | 2.07 | 0.001 |
| -9.00 | 1.59 | 1.21 | 2.08 | 0.001 |
| -8.50 | 1.65 | 1.26 | 2.16 | 0.000 |
| -8.00 | 1.68 | 1.28 | 2.20 | 0.000 |
| -7.50 | 1.68 | 1.27 | 2.21 | 0.000 |
| -7.00 | 1.62 | 1.23 | 2.14 | 0.001 |
| -6.50 | 1.66 | 1.25 | 2.21 | 0.000 |
| -6.00 | 1.64 | 1.23 | 2.19 | 0.001 |
| -5.50 | 1.49 | 1.11 | 2.00 | 0.008 |
| -5.00 | 1.50 | 1.11 | 2.02 | 0.008 |
| -4.50 | 1.41 | 1.04 | 1.92 | 0.029 |
| -4.00 | 1.39 | 1.01 | 1.92 | 0.041 |
| -3.50 | 1.41 | 1.02 | 1.96 | 0.037 |
| -3.00 | 1.32 | 0.94 | 1.85 | 0.108 |
| -2.50 | 1.26 | 0.88 | 1.80 | 0.200 |
| -2.00 | 1.28 | 0.89 | 1.85 | 0.187 |
| -1.50 | 1.22 | 0.83 | 1.79 | 0.316 |
| -1.00 | 1.24 | 0.84 | 1.85 | 0.283 |
| -0.50 | 1.17 | 0.77 | 1.77 | 0.457 |
| 0.00 | 1.19 | 0.78 | 1.83 | 0.423 |
