# Supplementary material for: Association between severe lumbar disc degeneration and end-stage hip or knee osteoarthritis requiring joint replacement surgery: a population-based cohort study with a 26-year follow-up
Source: Arch Orthop Trauma Surg. 2025 May 12;145(1):288. doi: 10.1007/s00402-025-05908-7 (PMC12069494; doi:10.1007/s00402-025-05908-7)
Supplement: Supplementary file 4 — Supplementary Material 4 [file 402_2025_5908_MOESM4_ESM.docx]

**Supplementary Table 4. The distribution of degeneration severity at different vertebral levels**

| \| Vertebral level \| Non-severe degeneration \| Severe degeneration \| \| --- \| --- \| --- \| \| L1-L2 \| 1094 (94.9%) \| 59 (5.1%) \| \| L2-L3 \| 1063 (92.2%) \| 90 (7.8%) \| \| L3-L4 \| 1073 (93.1%) \| 80 (6.9%) \| \| L4-L5 \| 1002 (86.9%) \| 151 (13.1%) \| \| L5-S1 \| 857 (74.3%) \| 296 (25.7%) \| \|  \|  \|  \| \| Total discs \| 5089 (88.3%) \| 676 (11.7%) \| \| L1-S1 mean degeneration \| 848 (73.5%) \| 305 (26.5%) \| |
| --- | --- | --- | --- | --- | --- | --- | --- | --- | --- | --- | --- | --- | --- | --- | --- | --- | --- | --- | --- | --- | --- | --- | --- | --- | --- | --- | --- |
