# Supplementary material for: Association between severe lumbar disc degeneration and end-stage hip or knee osteoarthritis requiring joint replacement surgery: a population-based cohort study with a 26-year follow-up
Source: Arch Orthop Trauma Surg. 2025 May 12;145(1):288. doi: 10.1007/s00402-025-05908-7 (PMC12069494; doi:10.1007/s00402-025-05908-7)
Supplement: Supplementary file 5 — Supplementary Material 5 [file 402_2025_5908_MOESM5_ESM.docx]

**Supplementary Table 5. Diagnosis or indication related to MRI (N=1,153)**

| Diagnosis | Frequency | L1-L5 mean  deg. grade | SD | L1-L5 severe degeneration (%) |
| --- | --- | --- | --- | --- |
| Spinal stenosis | 531 (46.1%) | 3.74 | 0.42 | 169 (31.8%) |
| Lumbar and other intervertebral disc disorders with radiculopathy | 126 (10.9%) | 3.61 | 0.36 | 22 (17.5%) |
| Other | 107 (9.3%) | 3.58 | 0.45 | 27 (25.2%) |
| Low back pain | 73 (6.3%) | 3.53 | 0.36 | 10 (13.7%) |
| Low back pain with sciatica | 52 (4.5%) | 3.60 | 0.37 | 14 (26.9%) |
| Other intervertebral disc degeneration | 49 (4.2%) | 3.62 | 0.34 | 5 (10.2%) |
| Spondylolisthesis | 37 (3.2%) | 3.71 | 0.40 | 12 (32.4%) |
| Lower limb pain or arthrosis | 34 (2.9%) | 3.63 | 0.45 | 11 (32.4%) |
| Spondylosis without myelopathy or radiculopathy, lumbosacral region | 26 (2.3%) | 3.66 | 0.37 | 8 (30.8%) |
| Other cancer | 21 (1.8%) | 3.49 | 0.40 | 5 (23.8%) |
| Rheumatic disease | 16 (1.4%) | 3.61 | 0.40 | 3 (18.8%) |
| Sciatica, unknown etiology | 16 (1.4%) | 3.53 | 0.39 | 4 (25.0%) |
| Vertebral fracture | 13 (1.1%) | 3.54 | 0.38 | 1 (7.7%) |
| Breast cancer | 11 (1.0%) | 3.69 | 0.39 | 3 (27.3%) |
| Radiculopathy | 8 (0.7%) | 3.60 | 0.49 | 3 (37.5%) |
| Difficulty in walking | 8 (0.7%) | 3.48 | 0.18 | 3 (37.5%) |
| Dorsalgia, unspecified | 8 (0.7%) | 3.40 | 0.65 | 0 (0.0%) |
| Multiple myeloma | 7 (0.6%) | 3.63 | 0.31 | 1 (14.3%) |
| Other spondylosis with radiculopathy | 5 (0.4%) | 4.00 | 0.68 | 3 (60.0%) |
| Spondylolysis | 5 (0.4%) | 3.64 | 0.26 | 1 (20.0%) |
