# Supplementary material for: Association between severe lumbar disc degeneration and end-stage hip or knee osteoarthritis requiring joint replacement surgery: a population-based cohort study with a 26-year follow-up
Source: Arch Orthop Trauma Surg. 2025 May 12;145(1):288. doi: 10.1007/s00402-025-05908-7 (PMC12069494; doi:10.1007/s00402-025-05908-7)
Supplement: Supplementary file 6 — Supplementary Material 6 [file 402_2025_5908_MOESM6_ESM.docx]

**Supplementary Table 6. Arthroplasty indications from the Care Register for Health Care (CRHC) and Finnish Arthroplasty Register (FAR)**

| Diagnosis (TKA) | Frequency |
| --- | --- |
| Primary knee osteoarthritis | 281 (94.0%) |
| Other | 11 (3.7%) |
| Secondary hip osteoarthritis | 6 (2.0%) |
| Other arthritis | 1 (0.3 %) |
| Total (TKA) | 299 |

| Diagnosis (THA) | Frequency |
| --- | --- |
| Primary hip osteoarthritis | 141 (82.9%) |
| Other | 18 (10.6%) |
| Secondary hip osteoarthritis | 6 (3.5%) |
| Rheumatoid arthritis | 5 (2.9%) |
| Total (THA) | 170 |
